# Polymorphism of *PfATPase *in Niger: detection of three new point mutations

**DOI:** 10.1186/1475-2875-8-28

**Published:** 2009-02-18

**Authors:** Maman Laminou Ibrahim, Nimol Khim, Hassane Hadiza Adam, Frédéric Ariey, Jean-Bernard Duchemin

**Affiliations:** 1 Centre de Recherche Médicale et Sanitaire (CERMES), BP 10887 Niamey, Niger; 2 Unité d'épidémiologie moléculaire, Institut Pasteur du Cambodge, 5 Boulevard Monivong, BP 983, Phnom Penh, Cambodia

## Abstract

**Background:**

*Plasmodium falciparum *resistance to drugs remains a major public health issue in Niger. The therapeutic failure index for chloroquine and sulphadoxine-pyrimethamine are, respectively 20% and 21.9%. In December 2005, the National Malaria Control Programme promoted the use of artemisinin combination therapy (ACT) as first-line treatment of the uncomplicated malaria cases. Recently, studies have shown a relationship between the SERCA *PfATPase6 *gene and artemisinin efficacy, and pointed it out as a potential molecular marker for resistance. The goal of this work was to describe the baseline polymorphism of *PfATPase6 *gene in Niger, at a time when the national implementation of the ACT policy had just begun.

**Materials and methods:**

The DNA polymorphism of the *PfATPase6 *gene of 87 *P. falciparum *samples from Niger was analysed by sequencing. The links between the mutation occurrence and environment and human host factors were tested by bivariate analysis.

**Results:**

The *P. falciparum PfATPase6 *gene presented polymorphisms at codons 537, 561, 569, 630, 639, 716 levels. All the mutations found were rare, except the *PfATPaseN569K *found in 17.2% of samples. No associated factor has been observed.

**Conclusion:**

The *P. falciparum PfATPase *gene is polymorphic at the 569 codon. As ACT is getting more and more used, the *PfATPase6 *gene polymorphism needs to be monitored in association with phenotypic – *in vivo *and/or *in vitro *– drug efficacy tests.

## Background

For many diseases, the history of anti-infectious drugs shows us that the resistance to treatment does appear after a more or less long time. This is the case for HIV, tuberculosis and, in this case, malaria [1]. The World Health Organization (WHO) recommends the use of therapeutic combination with artemisinin derivatives for a better efficacy and to delay the occurrence of resistance by exploiting the synergic and/or additive properties of the constituent drugs [2]. The *pfATPase *is an ATPase-dependent ionic pump from the *Plasmodium *endoplasmic reticulum membrane related to cations transport. The artemisinin and derivatives should negatively regulate this cationic pump [3]. Until recently, only six SNPs of PfATPase were known [4,5]. The *pfATPase6S769N *mutation has been found strongly associated with artemether high IC50s in French Guyana [4] and has been proposed as a molecular marker of resistance against artemisinin. The *Xenopus *model [6] and docking simulation [7] suspected the 263 codon to be involved in the binding of artemisins to PfATPase6. Noting that this mutation site was never found involved in field studies, Data regarding sequences of African samples reported several mutation points: Jambou and *et al *[4] and Ferreira *et al *[5], respectively, sequenced 16 Senegalese samples and eight samples from Sao Tomé. Cojean *et al *[8] reported sequencing partial results of 154 samples, mainly from West Africa. From these three studies, only six mutation points were found. Recently, Dhalstrom *et al *[9] added the results of sequences of 345 East African and 10 West African samples, increasing the number of known SNPs to 33. Among these, three SNPs were found with a higher than 5% percentage of mutations: the codons H431K, N569K and A630S.

In Niger, the resistance of *Plasmodium falciparum *to treatment is a major public health issue. At the beginning of this century, a 20% therapeutic failure index for chloroquine has been found among children under five years of age [10,11]. Molecular monitoring of treatment efficacy showed 45.7% of *pfcrtK76T *and 60.2% of *pdhfrS108N *[12] at the Niamey National Hospital. In the Niger Valley, these molecular markers turned out having respective prevalences of 50.8% and 57.7% [13]. Furthermore, a DNA-microarray study, targeting 34 *P. falciparum *resistance-associated SNPs and including *PfATPase *codons, showed a clear increase of resistance to chloroquine and to sulphadoxine/pyrimethamine over a four-years period [14]. In December 2005, Niger has switched as its first-line treatment policy for uncomplicated cases of malaria to the use of artemisinin-based combination therapy (ACT). Having started in 2006, the implementation has now reached the whole country. The main goal of the study, performed at a time when the national implementation of the ACT policy had just begun, was to describe the genetic polymorphism of the *pfATPase6 gene*. This should provide useful baseline data for the malaria resistance monitoring network (RSRA), [13] set-up in Niger in 2002 and included in the framework of the West African malaria resistance survey network, RAOTAP II.

## Methods

### Samples

Ninety two *P. falciparum *microscopy-positive samples from asymptomatic carriers have been tested. They were all coming from two villages of Sahel in south-west Niger. The two villages differed by the duration of the malaria transmission: short (two to three months) in Banizoumbou and longer (six months) in Zindarou. The samples were stratified by age (younger than 10 years old and equal and older than 10 years old), by village (Banizoumbou/Zindarou), by year (2003/2006) and by parasitaemia classes (below 1,000 parasites/μl and above 1,000 parasites/μl). Ethical clearance was obtained from the National Ethics Committee of Niger.

### DNA extraction

The DNA from the finger prick blood dried on filter cards was extracted in 96 well-plates by resin (Instagen). Briefly, dried red blood cells were first treated by lysis buffer HBS1%/saponine 10% (HEPES 100mM, NaCl 1.4M, KCl 100mM) and washed three times. The parasites pellets have been heated (56°C then 90°C) with 200 μl of Instagen resin. Finally the DNA was purified by centrifugation during 20 minutes.

### PCR amplification and sequencing

A nested PCR allowed the amplification of a portion of the *PfATPase *gene. The primary PCR allows the production of 896 pb amplicon with primers P17.1 for 5'-TGGATCAATAATACCTAATCCACCTA-3' and P17.1rev: 5'-AATATTGTTATTCAGA ATATGATTAT AA-3'. The nested PCR with internal primers P17 for 5'-AGCAAATATT TTCTGTAACGATAATA-3' and P17 rev 5'-TGTTCTAATTTATAATAATCATCTGT-3' amplified a 798 pb amplicon which was sequenced in the South Korea Biotechnology Institute. Most of the mutations found in field samples were described in that zone, including codons for the cytoplasmic domain of the *PfATPase6 *[15].

### Analysis

Data were included in a data file with EPI-INFO v. 6.0 and bivariate relations were analysed by the Chi-square or Fisher exact tests.

## Results

### Samples characteristics

Fifty percent of the 92 samples came from Zindarou and the other half from Banizoumbou. The mean age was 13 years old and the F/M sex ratio was 1.35. The mean parasitaemia was 2,681 parasites/μl.

### Genetic polymorphism and global prevalence of mutations

Sequencing was successful in 87 samples. Genetic polymorphism was found at six codons of the *PfATPase *gene: 537, 561, 569, 630, 639 and 716. The mutation at 537 codon did not lead to any potential protein change. The five others led to protein change. Among these, two are newly described: K561N and K716R. The prevalence of the different mutations at the 537, 561, 630, 639, and 716 codons was only 1.1% (1/87). The mutation *PfATPase N569K *prevalence was 17.2% (Table 1). Only one sample was carrying two mutations: N569K and G639D.

**Table 1:** SNPs codons and occurrences

**SNPs**	**Codons**	**%**
*PfATPaseD537D*	GAC-GAT	1.1% (N = 87)
*PfATPase K561N*	AAA-AAT	1.1%(N = 87)
*PfATPaseN569K*	AAT-AAA	17.2% (N = 87)
*PfATPaseA630S*	GCT-TCT	1.1% (N = 87)
*PfATPaseG639D*	GGC-GAC	1.1%(N = 87)

### Bivariate relation of *PfATPase N569K *according to parasitaemia, age, village and year

Neither the parasitaemia, nor the sex, nor the age, nor the villages, nor the collection years were related to the *PfATPase N569K *mutation.

## Discussion

This study describes for the first time a *PfATPase6 *gene sequence polymorphism in Niger. It increases the number of West African samples sequences and mutations points, adding three new mutations points: 537, 561 and 716. Among the six different observed mutations, only one, the *N569K *mutation, was relatively frequent – 17.2% of isolates. Dahlstrom *et al *[9] had also found a high prevalence of this mutation in Zanzibar (36%) and Tanzania (29%). This codon was out of the Menegon *et al *[16] studied gene zone, as were the two new found 357 and 561 mutations. The *pfATPaseS769N *mutation was absent in these Niger samples, as it had been shown in China [17] and in Tanzania tested either by DNA-microarrays [18] or by sequencing [9]. The *pfATPaseS769N *mutation has been recently described as a potential molecular marker for *P. falciparum *resistance to artemisinin [4]. The quasi-absence of this mutation in African samples [19] – since its first report, it had only been found once by Cojean *et al *[8] – did not disprove this hypothesis. Recently, a study from Menegon *et al *[16], carried out a polymorphism study of *PfATPase *in African isolates at codons 402 and 431, which the present work did not target. In the Niger study, there was no correlation between this mutation and other factors, such as village, age or time of collection. These factors have been correlated with chloroquine and pyrimethamine resistance in previous work [14]. The same previous study checked for five *PfATPase*6 gene SNPs (538, 574, 623, 683 and 769) by DNA-microarrays [20]. By DNA-microarrays, the *pfATPaseA623E *mutation was found in 4.7% of the Niger samples [14], but sequencing did not confirm this.

The present work showed six different mutations for 87 samples of Niger, while Dahlstrom *et al *found 31 among 302 samples in Zanzibar and three among 39 Tanzanian samples [9]. Menegon *et al *found seven mutations sites for 71 specimens from west, central and south-east Africa [16], including Madagascar. All these figures are comparable and, in a given geographical zone, such molecular variation has to be linked with the sampling effort, the number of samples, but also of sequenced base pairs in a given genomic area.

## Conclusion

The present work did not show the S769N mutation, presently candidate as a molecular marker for artemether resistance. However, the molecular diversity of *PfATP6 *seems more pronounced than previously demonstrated. The variable genetic background needed for the artemisinin-driven selection of resistant variants could be present in Africa. At a time when almost all African countries have adopted artemisinin derivatives as first-line treatment, the molecular monitoring of the *PfATP6 *gene is of prior importance. The baseline data established before the ACT implementation will support ulterior comparison. Among the highlighted mutations, the *N569K *mutation presents notable occurrences both in West and East Africa. Future functional studies, such as docking simulation and binding affinity, may be useful for prediction of the impact of the diverse mutations and the design of future molecular monitoring tools supporting the national control programmes.

## Competing interests

The authors declare that they have no competing interests.

## Authors' contributions

MLI carried out the molecular genetic studies and drafted the first manuscript version. HHA participated to field work and DNA extraction. NK participated in the sequence alignment. FA and JBD conceived the study, participated in its design and coordination and elaborated the final version of manuscript. All authors read and approved the final manuscript.
